# Ectopic gallbladder with congenital biliary dilatation: a pediatric case report

**DOI:** 10.1186/s40792-022-01401-0

**Published:** 2022-03-26

**Authors:** Kanako Omata, Mariko Yoshida, Kan Suzuki, Hiroshi Kawashima, Jun Fujishiro

**Affiliations:** 1 Department of Pediatric Surgery, Graduate School of Medicine, The University of Tokyo, 7-3-1, Hongo, Bunkyo-ku, Tokyo, 113-8654 Japan; 2 Department of Pediatric Surgery, Saitama Children’s Medical Center, Saitama, Japan

**Keywords:** Ectopic gallbladder, Congenital biliary dilatation, Pediatric

## Abstract

**Background:**

An ectopic gallbladder is a rare anomaly and can result in the misinterpretation of imaging findings and clinical confusion. Knowledge of such anomalies facilitates accurate diagnoses and prompt management. We report a pediatric case of an ectopic gallbladder concomitant with congenital biliary dilatation (CBD).

**Case presentation:**

A 9-year-old girl was referred to our hospital for elevated liver enzyme levels. Following physical examination and a review of medical imaging findings, she was diagnosed with Todani type IV-A CBD. We could not visualize the gallbladder by abdominal ultrasonography, CT, and MRI scans; therefore, we suspected gallbladder agenesis. A laparoscopic excision of the extrahepatic bile duct was performed to treat the CBD. Neither a gallbladder nor a cystic duct were revealed on the liver undersurface. Therefore, gallbladder agenesis was considered as a diagnosis based on preoperative imaging and intraoperative findings. However, during dissection of the hepatic hilum, a cyst-like structure was found on the ventral side of the common hepatic duct, slightly to the right, and a small additional duct that originated from the cystic structure was found. Upon incision, a small amount of bile was drained from the small duct. Thus, the cystic structure was diagnosed as an ectopic gallbladder with hypoplasia. Following the removal of the ectopic gallbladder, the extrahepatic bile duct was excised. Subsequently, laparoscopic Roux-en-Y hepaticojejunostomy was performed without any complications. Postoperative histopathological evaluations of the resected specimen revealed Rokitansky–Aschoff sinuses in the resected cystic lesion. The pathological investigations confirmed the diagnosis of an ectopic gallbladder. Following an uneventful postoperative course, the patient was discharged on day nine.

**Conclusions:**

To our knowledge, this is the first pediatric case report describing an ectopic gallbladder concomitant with CBD. If the gallbladder cannot be detected in a preoperative imaging examination, it is important to consider the possibility of an ectopic gallbladder.

## Background

An ectopic gallbladder is a rare anomaly of the gallbladder with an incidence rate of 0.1–0.7% [1] and can result in the misinterpretation of imaging findings, leading to clinical confusion [2]. It can also lead to surgical confusion and an increased risk of morbidity in hepatobiliary surgery if not detected preoperatively or intraoperatively. Knowledge of rare gallbladder anomalies facilitates accurate diagnoses and prompt management.

Herein, we report a pediatric case of ectopic gallbladder concomitant with congenital biliary dilatation (CBD).

## Case presentation

**Fig. 1:**
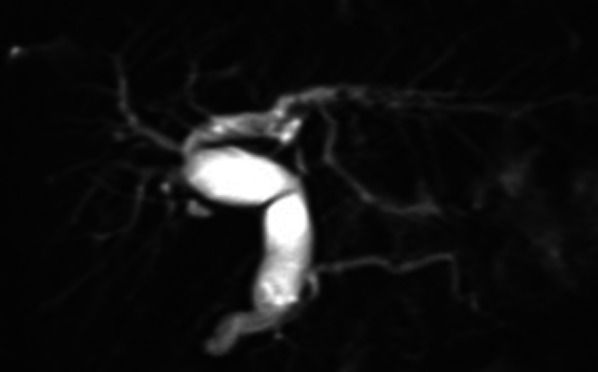
Preoperative magnetic resonance cholangiopancreatography (MRCP). MRCP showing dilatation of bile duct from the left and right intrahepatic bile ducts to the common bile duct. The gallbladder is not detected on MRCP

**Fig. 2:**
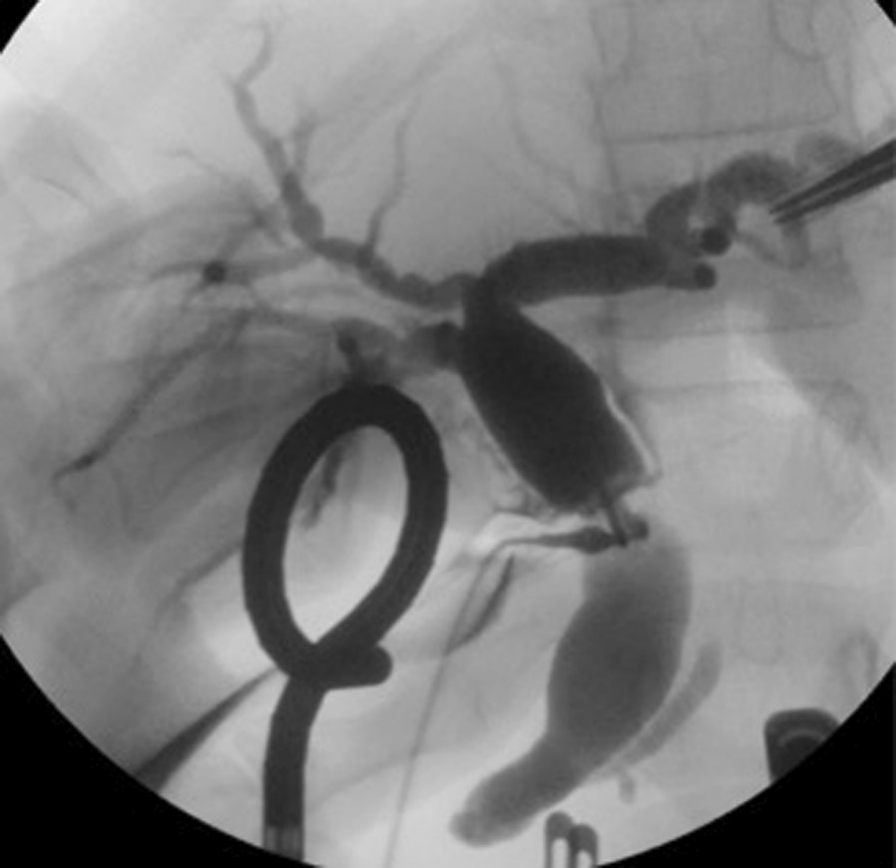
Intraoperative cholangiography. Intraoperative cholangiography performed by direct puncture of the dilated common bile duct does not show the gallbladder or the cystic lesion in the hepatic hilum. The anterior and posterior segment branches separately join the common hepatic duct

**Fig. 3:**
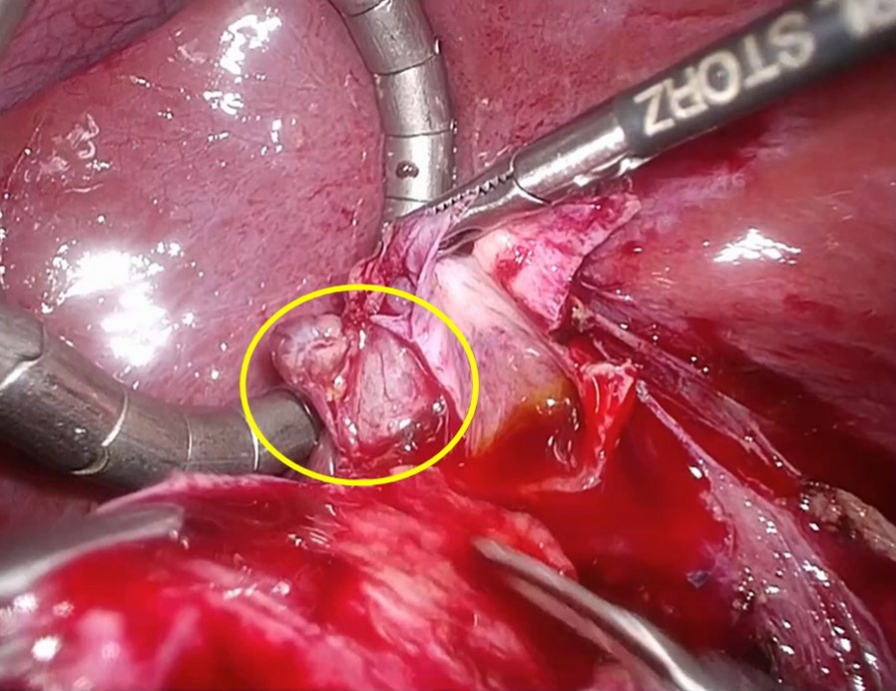
Intraoperative findings. A cystic structure on the ventral side of the common hepatic duct, slightly to the right (circle), is diagnosed as an ectopic gallbladder during dissection of the common hepatic duct. The distance between the cystic duct and bifurcation is estimated to be 1.5 cm. The ectopic gallbladder is resected

**Fig. 4:**
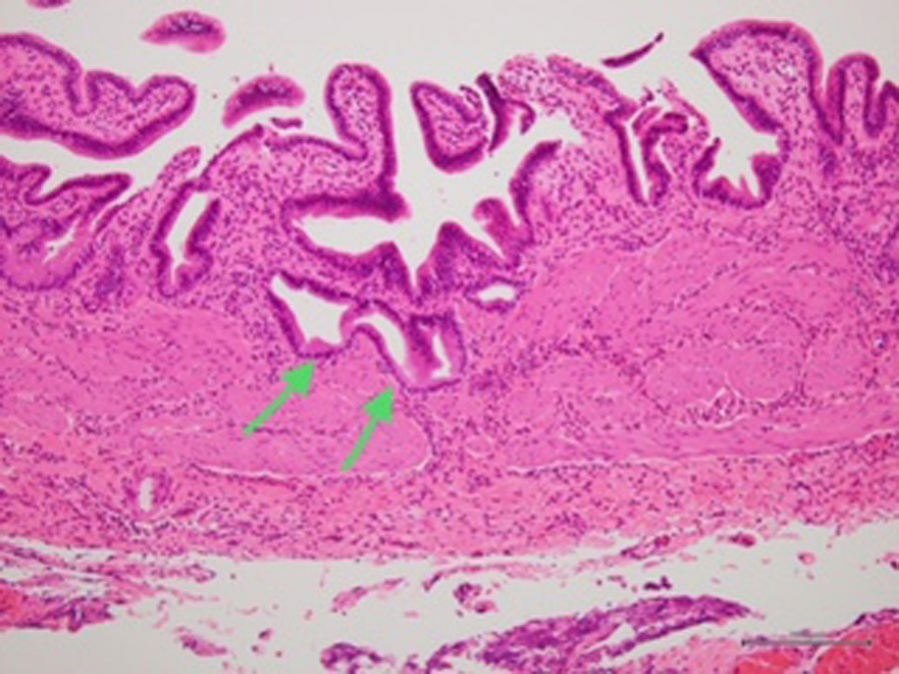
Histopathological findings of the gallbladder. The Rokitansky–Aschoff sinus (arrows) is identified in the wall of the cystic structure. The pathological diagnosis of the cystic structure is of an ectopic gallbladder

A 9-year-old girl was referred to our hospital for further examination regarding dilatation of the common bile duct, which was found by abdominal ultrasonography performed for the evaluation of elevated liver enzyme levels. Magnetic resonance cholangiopancreatography (MRCP) revealed dilated bile ducts from the left and right intrahepatic bile ducts to the common bile duct, and the patient was diagnosed with Todani type IV-A CBD (Fig. 1). The gallbladder was not detectable by abdominal ultrasonography, computerized tomography or MRCP; therefore, we suspected gallbladder agenesis preoperatively. The patient underwent laparoscopic excision of the extrahepatic bile duct to treat the CBD. Initial laparoscopic exploration did not reveal a gallbladder or cystic duct on the liver undersurface. Therefore, we considered gallbladder agenesis based on both preoperative imaging and intraoperative findings. Intraoperative cholangiography was performed by direct puncture of the common bile duct, and it did not show the gallbladder and bile duct (Fig. 2). However, during dissection of the hepatic hilum, we detected a cystic structure on the ventral side of the common hepatic duct, slightly to the right, and the presence of a small additional duct that originated from the cystic structure (Fig. 3). The size of the cystic structure was estimated to be 3 cm and the distance between the cystic duct and bifurcation was estimated to be 1.5 cm. A small amount of bile was drained upon incising the small duct. Thus, we diagnosed the cystic structure as an ectopic gallbladder with hypoplasia. After the ectopic gallbladder was removed, an excision of the extrahepatic bile duct followed by laparoscopic Roux-en-Y hepaticojejunostomy was completed without any complications. Postoperative histopathological evaluations of the resected specimen revealed Rokitansky–Aschoff sinuses in the resected cystic lesion (Fig. 4). Findings from the pathological investigations confirmed the diagnosis of an ectopic gallbladder. The patient’s postoperative course was uneventful, and she was discharged on the ninth postoperative day. She recovered without any hepatobiliary complication 10 months after the operation.

## Discussion

Gallbladder anomalies vary, ranging from variations in size, site, anomalies of the duct system, and shape. Among these anomalies, an ectopic gallbladder, caused by a variation in location, is the most common [2]. The most common ectopic locations are (1) under the left hepatic lobe, (2) intrahepatic, (3) transverse, and (4) retroplaced (retrohepatic or retroperitoneal) [3]. In our case, the ectopic gallbladder was located on the ventral side of the common hepatic duct, and to the best of our knowledge, this location has not been reported previously.

There are several ways in which an ectopic gallbladder can lead to clinical uncertainty. For example, an ectopic gallbladder located away from the peritoneum may not instigate the typical peritoneal symptoms associated with acute cholecystitis, thereby delaying the diagnosis [4, 5]. However, the condition may be complicated, given that a floating gallbladder may also be predisposed to torsion, particularly if provoked by peristaltic action of nearby bowel segments. Moreover, ectopic gallbladders located beneath the left hepatic lobe are susceptible to herniation through the foramen of Winslow. For these reasons, it has been suggested that an ectopic gallbladder should be resected when found [6].

Ultrasonography, computed tomography, and radionuclide imaging are common modalities used for imaging-based diagnosis of an ectopic gallbladder [7]. However, an ectopic gallbladder can be left undetected on imaging studies, particularly in patients with a contracted gallbladder or in those with an incompletely distended gallbladder secondary to inadequate preoperative fasting. In our case, the ectopic gallbladder was small and located beside the common hepatic duct at the hilum of the liver; therefore, it could not be detected by preoperative examination. Ectopic gallbladders are usually diagnosed intraoperatively. Intraoperative complications are avoidable if the ectopic gallbladder is recognized early in the operation. However, delay in recognition of an ectopic gallbladder during surgery may lead to biliary complications, such as gallbladder rupture or biliary peritonitis [3].

Conversely, gallbladder agenesis, which we suspected preoperatively in our case, is another rare congenital anomaly with an incidence rate of 0.1–0.65%, and approximately 50% of these patients present with symptoms of biliary diseases [8]. The developmental abnormality of the biliary system results in an ectopic gallbladder and gallbladder agenesis. The liver, gallbladder, and biliary system begin to develop early in the fourth week of intrauterine life in the form of a ventral outgrowth from the caudal part of the foregut. This hepatic diverticulum is divided into two parts as it grows: one representing the primordium of the liver and the other representing the primordium of the gallbladder and cystic duct. By the seventh week of intrauterine life, vacuolation occurs, and the gallbladder and the cystic duct develop a lumen [9]. Failure of this developmental process at any stage results in agenesis of the gallbladder, whereas inappropriate migration of the gallbladder primordium results in an ectopic gallbladder [10]. In our case, the patient would have been diagnosed with gallbladder agenesis and the ectopic gallbladder would not have been detected had she not undergone surgery for CBD. Similarly, some previous reports of gallbladder agenesis might include cases with an ectopic gallbladder.

An ectopic gallbladder has been reported to be associated with congenital diseases such as congenital agenesis of the right lobe of the liver or duplication of the common bile duct [11, 12]; however, to the best of our knowledge, the association between an ectopic gallbladder and CBD has not been reported previously. Congenital biliary dilation is occasionally associated with an anomalous biliary tree. Specifically, an anomalous biliary tree may not be easily identified on preoperative imaging. Therefore, surgeons should be mindful of the possibility of anatomical abnormalities of the biliary tree during hepatobiliary surgery. In our case, while we suspected the absence of the gallbladder until halfway through the operation, meticulous exploration at the hepatic hilum revealed an ectopic gallbladder.

## Conclusions

To the best of our knowledge, this is the first pediatric case report describing an ectopic gallbladder concomitant with CBD. It is important to consider the possibility of an ectopic gallbladder when the gallbladder is not identified by preoperative imaging for hepatobiliary surgery.

## Data Availability

The datasets supporting the conclusions of this article are included within the article.

## Abbreviations

CBD: Congenital biliary dilatation
MRCP: Magnetic resonance cholangiopancreatography

## References

[1] Gu W, Tong Z (2020). Ectopic gallbladder: a case report and review of the literature. Asian J Surg.

[2] Mohammed AA, Arif SH (2019). Midline gallbladder makes a challenge for surgeons during laparoscopic cholecystectomy; case series of 6 patients. Ann Med Surg (Lond).

[3] Revzin MV, Scoutt L, Smitaman E, Israel GM (2015). The gallbladder: uncommon gallbladder conditions and unusual presentations of the common gallbladder pathological processes. Abdom Imaging.

[4] Chung CC, Leung KL, Lau WY, Li AK (1997). Ectopic gallbladder revisited, laparoscopically: a case report. Can J Surg.

[5] Popli MB, Popli V, Solanki Y (2010). Ectopic gallbladder: a rare case. Saudi J Gastroenterol.

[6] Guerin JB, Venkatesh SK, Roberts LR (2015). Ectopic gallbladder. Clin Gastroenterol Hepatol.

[7] Rafailidis V, Varelas S, Kotsidis N, Rafailidis D (2014). Two congenital anomalies in one: an ectopic gallbladder with Phrygian cap deformity. Case Rep Radiol.

[8] Tagliaferri E, Bergmann H, Hammans S, Shiraz A, Stüber E, Seidlmayer C (2016). Agenesis of the gallbladder: role of clinical Suspicion and magnetic resonance to avoid unnecessary surgery. Case Rep Gastroenterol.

[9] Ando H (2010). Embryology of the biliary tract. Dig Surg.

[10] Fiaschetti V, Calabrese G, Viarani S, Bazzocchi G, Simonetti G (2009). Gallbladder agenesis and cystic duct absence in an adult patient diagnosed by magnetic resonance cholangiography: report of a case and review of the literature. Case Rep Med.

[11] Bender EA, Springhetti S, Shemisa K, Wittenauer J (2007). Left-sided gallbladder (sinistroposition) with duplication of the common bile duct. J Soc Laparoendosc Surg.

[12] Boufettal R, Khaiz D, Jai SR, Chehab F, Bouzidi A (2008). Right liver agenesis with ectopic gallbladder and bile duct calculi. Gastroenterol Clin Biol.

